# The multi-level ammoniation on the digestibility of palm press fiber

**DOI:** 10.5455/javar.2021.h507

**Published:** 2021-06-19

**Authors:** Armina Fariani, Anggriawan Naidilah Tetra Pratama, Gatot Muslim

**Affiliations:** Animal Science Department, Sriwijaya University, Kota Palembang, Indonesia

**Keywords:** Multilevel ammoniation, digestibility, palm press fiber, PPF

## Abstract

**Objective:**

This research aimed to study the multi-level ammoniation on the digestibility of palm press fiber (PPF) through *in vitro* methods.

**Materials and Methods:**

This research was determined using a complete randomized design of three ammoniation treatments on PPF with three replications: (1) untreated PPF (Con), (2) ammonia treatment 4% (A-4), and (3) ammonia multi-levels of 6%, 4%, and 2% (A-Mul).

**Results:**

The results showed a significant effect on the chemical composition of feed in the A-Mul treatment compared to Con (*p* < 0.05) and a non-significant effect when compared to the A-4 treatment. Overall, the content of crude protein, ether extract, and crude fiber in the A-Mul treatment increased. Except in the dry matter (DM), neutral detergent fiber and acid detergent fiber decreased. The results of the *in vitro* analysis showed an increase in digestibility of DM, Organic matter digestibility, N-NH_3_, and volatile fatty acids (VFA) in ammoniation treatment. N-NH_3_ and VFA showed non-significant differences between treatments A-4 and A-Mul (*p* > 0.05).

**Conclusion:**

Multi-level ammoniation has affected the loosening of the fiber fraction bonding in the PPF, thereby changing the value of the chemical composition and the digestibility of PPF. Multi-level ammonia can be used as an alternative to processing high-fiber feed.

## Introduction

The high activity of land use for industrial activity, housing, and transportation sectors has become a problem in the supply of raw materials for ruminant animal feed, resulting in the lack of forage feed having good nutrient quality. Several studies have shown that competition for land use in an area can occur with an increase in human population and food needs, which impacts the decreasing livestock population due to the reduced availability of forages for a ruminant [1,2]. On the other hand, Indonesia, as one of the countries with crop productivity in the plantation sector, such as the palm oil industry, has increased significantly every year. This impacts the increasing industrial byproducts such as midribs, leaves, and stems [3]. Meanwhile, the byproducts of palm oil processing are empty bunches, palm kernel cake, solid mud, and palm press fiber (PPF) [4].

PPF is one of the byproducts of palm fruit extraction. In a study conducted by Mathius et al. [5], the PPF could be used as an animal feed with a potential value of producing 1one ha of oil palm plantation at 2,681 kg/dry matter (DM)/year. So, if in 2019, Indonesia has 8 million ha (90% in production conditions), it will produce 21.448 million/ton/DM/year [6]. Abundant PPF can be used as an alternative feed continuously and does not depend on the season. However, PPF has a low crude protein (CP) content and high crude fiber (CF) content, which is a limiting factor for its use as animal feed [7,8], but the nutritional quality of PPF can still be improved by chemical treatment, e.g., ammoniation.

Ammoniation techniques can improve the quality of PPF nutrition to benefit livestock and increase CP levels. The CP content is determined from ammonia in urea which plays a role in loosening CF [9]. Furthermore, it results in increased digestibility and CP content by absorbing nitrogen into the feed [10-12]. This processing, in principle, uses urea as a source of ammonia which is mixed into the PPF. Ammonia fiber expansion technology, which has been widely used in the world today, uses high pressure and temperature. In Indonesia, this cannot be carried out due to increased production costs and knowledge gaps among farmers [13,14].

The ammoniation process refers to the straw ammoniation procedure carried out under anaerobic conditions with an optimum curing time of 3–4 weeks or even 6–8 weeks, depending on the ambient temperature [9]. The large amount of time needed to process ammoniation with urea is a weak factor in this method. The reason for the long time required is due to the process of converting urea to ammonia. Furthermore, the ammonia produced causes changes in the composition and structure of the cell wall by loosening the bonds between lignin, cellulose, and hemicellulose. Chemical reactions that occur (by cutting hydrogen bridges) cause tissue expansion and increase cell wall flexibility to facilitate penetration (breakthrough) by cellulase enzymes produced by microorganisms [10,15,16]. The length of curing time is an obstacle in ammonia so that the supply of feed for livestock is limited. To answer the challenges of livestock feed needs and to overcome the problem of the length of curing time, an alternative process for processing the PPF with multi-level ammonia treatment is carried out. Multi-level ammoniation is an ammonia processing technique carried out in stages with different urea levels to shorten the curing time. The feed can be used quickly, has good nutritional quality, and is easily applied to the livestock industry.

Based on the description above, in this study, PPF processing was carried out with chemical treatment in the form of multi-level ammonia treatment with a concentration of urea at 6%, 4%, and 2% in the specified time for 14 days to shorten the curing time.

## Materials and Methods

### Methods and sample preparation

This research was determined by using a complete randomized design of three ammoniation treatments on PPF, with each treatment consisting of three replications: (1) untreated PPF (Con), (2) ammonia treatment 4% (A-4), and (3) ammonia multi-level 6%, 4%, and 2% (A-Mul). The source of material for ammonia was urea fertilizer produced by PT PUSRI with 48% nitrogen content and 0.5% water content. Meanwhile, the PPF was obtained from the process of extracting oil palm fruits at PT Adira. 

### Ammoniation

In the process of ammoniation, PPF was divided into two stages. First, ammoniation with 4% urea concentration and ammoniation and then with urea concentrations of 6%, 4%, and 2%. Each treatment used urea levels accompanied by different curing times. Multi-level ammonia is a feed processing technology developed by focusing on shortening the curing period and increasing the digestibility value of feed with high fiber content. In the 4% urea treatment, the curing time was carried out for 4 weeks, whereas in multi-level urea, the shorter tiered curing time was 2 weeks.

The process of making urea ammoniation feed 4% begins by weighing 1 kg PPF and urea at 4% of the weight of the treatment PPF, which is 40 gm. The weighed urea is dissolved with 1 l of water to make an ammoniation solution, and is stirred until it is completely dissolved (homogeneous) and put in a spray tube. PPF that has been weighed is sprayed with ammoniation solution until it is distributed evenly. Then, it is put in a plastic bag consisting of two layers to prevent leakage or holes in the bag and to maintain anaerobic conditions during the curing period. Plastic bags used had a capacity of 3 kg and a thickness of 50 μm. After that, the plastic bag containing PPF + urea was tightly closed using a plastic strap which is then continued to make sure the situation inside the bag not have oxygen (anaerobic). Plastic bags were bound and a vacuum was placed at room temperature with a curing time for up to 4 weeks. After curing time, the bag was opened, and then the treatment feed was removed and dried first for 24 h before analysis in the laboratory.

Furthermore, for multi-level ammoniation in the preparation process of PPF and urea, the material is almost the same; the difference only lies in the composition/concentration of urea and curing time. The multi-level of ammoniation consists of three stages. Stage 1 starts with making ammoniation solution with a level of 6% urea with a curing time of 3 days. The results were dried for 24 h and then continued with stage 2, ammoniation with 4% urea concentration and 7 days curing time. The results were re-dried for 24 h and continued with stage 3 ammonia, with a level of 2% and a curing time of 3 days. Then, the last stage ammoniation results were dried for 4 h before analysis in the laboratory. Thus, the total time needed to make multi-level ammoniation was 14 days.

### Chemical analysis and in vitro incubation

Samples for measurement of DM, CP, CF, and ether extract (EE) were conducted according to the AOAC method [17] and fiber fraction analysis was conducted according to Van Soest’s analysis [18], while to measure the dry matter digestibility (DMD), N-NH_3_ and volatile fatty acids (VFA) were conducted according to *in vitro* Tiley and Terry’s method [19]. Before the *in vitro* process begins, the treatment feed is milled using a hammer mill, then refined using a 7 mm screen. Then, the filtered sample is weighed using digital analytical scales with as much as 1 g to be inserted into an *in vitro* tube labeled treatment code.

### Data analysis

The data acquired can be processed using Statistical Product and Service Solutions software program ver. 20. If there were differences between treatments, Duncan’s new multiple range test was tested.

## Results and Discussion

Based on the results of the analysis in Table 1, it was shown that the DM within the ammoniation treatment was considerably completely different from the control (*p* < 0.05) and not significantly different from the A-4. The decrease in DM content was thought to be due to the loss of water content in the sun-drying process. On the contrary, the CP content and EE in A-Mul increased significantly (*p* < 0.05) compared to other treatments. Increased CP content in the treatment feed ranged from 27% to 57%, and EE ranged from 9% to 26%. On the contrary, a significant decrease in CF content, neutral detergent fiber (NDF) and acid detergent fiber ADF (*p* < 0.05) occurred in the A-Mul treatment compared Con treatment, but it did not differ when compared with A-4.

Based on the analysis results in Table 2, it was shown that the DMD and organic matter (OM) digestibility (OMD) was considerably different from other treatments (*p* < 0.05). Meanwhile, VFA on A-Mul tended to show a considerable difference (*p* = 0.05) with the control treatment and not significantly different from the A-4 treatment. Furthermore, N-NH_3_ content showed significant results (*p* < 0.05) within the A-4 treatment compared to the Con treatment and was no different compared to the A-Mul.

DM is the basis used in animal feed to measure the quality of a feed ingredient. The higher the DM content of a feed ingredient, the higher the nutrient content contained in the feed. In general, a rise in DM content was followed by a decrease in the water content in the feed material. The results of the analysis that have been obtained indicate a reduction in DM on A-Mul, which can be seen in Table 1. The decrease in DM was suspected in A-Mul; there was a loosened bond on the cell wall caused by the impact of ammonia as a stable base inflicting cells from PPF to swell, as is the case with cookies that have been soaked in water for several h.

Furthermore, at the A-Mul process stage, the sun-drying process was carried out thrice. It causes the evaporation of ammonia compounds that bind with water in the cell wall caused by endothermic reactions because the ambient temperature is higher than the temperature of the solution. It will have an impact on decreasing the content of DM in the feed. According to Zhao et al. [16], the reduction in DM content in treatment feeds with various types of alkaline treatment in large feeds causes the release of water content in the feed due to the sun-drying process.

Changes in composition also occur in CP, EE, and CF and its fiber fractions. The increase occurred in the CP content and EE. The addition of urea, a complex compound containing nitrogen, causes an increase in CP content of feed ingredients. On the contrary, the increase in EE was thought to have been caused by an alkali process that loosened the cell wall structure, releasing ester compounds within the palm fiber content. This released because the PPF was a byproduct in the extraction to produce crude palm oil (CPO). With the remaining fat/ether content in the PPF, and through the process of ammoniation, the residual fat/ether content can be removed. The analysis of the EE content in the oil palm juice was obtained from small- and large-scale mills and showed a high composition ranging between 269 and 355 g/kg [7]. 

Table 1 shows the more urea used in ammoniation, the more CP content increased. The increased CP content was caused by urea in the ammonia process. The addition of urea in multi-level ammoniation with curing time for 12 days was known to optimally increase the CP content and the total N stored in the feed. The ammoniation process causes nitrogen (N) fixation into the feed cell tissue PPF, and this fixed nitrogen will be measured as CP. According to Adesogan et al. [9], with ammoniation treatment, it will enter cell tissues so that the protein content will increase due to fixed urea hydrolysis. Ammoniation treatment with urea has advantages compared to chemical treatments, such as NaOH, Ca(OH)2, and KOH, because of its ability to produce high N residues and increase CP levels in the ammoniation treatment [16,20,21].

**Table 1.** Chemical composition of ammoniated PPF.

Variable	Treatment		
	Con	A-4	A-Mul
DM% as fed	99.63^b^ ± 0.008	99.56^b^ ± 0.051	99.34^a^ ± 0.544
CP% of DM	3.742^a^ ± 0.632	6.263^ab^ ± 2.200	8.732^b^ ± 0.902
EE% of DM	12.36^a^ ± 0.803	14.86^b^ ± 0,156	16.39^c^ ± 0.108
CF% of DM	47.00^b^ ± 3.798	38.39^a^ ± 1.661	33.73^a^ ± 2.270
NDF% of DM	78.79^b^ ± 6.26	72.08^ab^ ± 6.15	66.40^a^ ± 4.76
ADF% of DM	60.69^b^ ± 5.69	49.51^ab^ ± 5.65	43.74^a^ ± 6.01

Con = untreated PPF; A-4 = urea 4%; A-Mul = urea (6%,4%,2%); DM = dry matter; CP = crude protein; EE = ether extract; CF = crude fiber; NDF = neutral detergent fiber; ADF = acid detergent fiber.

Means with different superscript letters in the same line differ significantly (*p *< 0.05).

**Table 2.** The effect of ammoniated PPF on the DMD, OMD, VFA, and N-NH_3_.

Item	Treatment		
	Con	A-4	A-Mul
DMD	44.75^a^ ± 2.12	52.00^a^ ± 4.06	73.10^b^ ± 3.39
OMD	25.50^a^ **± **9.84	29.93^a^ **± **7.57	47.09^b^ ± 4.04
TVFA	93.14^a^ ± 8.76	95.66^ab^ ± 14.32	114,89^b^ ± 5.12
N-NH_3_	3.74^a^ ± 0.17	5.16^b^ ± 0.82	4.27^ab^ ± 0.39

Con = untreated PPF; A-4 = urea 4%; A-Mul = urea (6%,4%,2%); DMD = dry matter digestibility; OMD = organic matter digestibility; TVFA = total volatile fatty acids; N-NH_3_ = ammonia.

Means with different superscript letters in the same line differ significantly (*p *< 0.05).

On the contrary, CF content, NDF, and ADF in the reduced treatment feed were shown to be due to the effect of ammoniation, except the ADF at A-4 treatment showed an increase. The CF content was depreciated due to swelling of the lignocellulose and lingo-hemicellulose bonds through three stages. First, there has been an absorption of alkaline reagents into cells that break the ester bonds among lignin, hemicellulose, and cellulose. Secondly, the breakdown of hydrogen bonds in the PPF, which causes cellulose crystallinity, was reduced, thereby loosening the physical structural parts. Third, reactions in the first and second stages result in a higher reaction area between rumen microbes and PPF, consequently making the rumen of microbes more easily digest structural carbohydrates from PPF [9,16,22].

Furthermore, the decrease in NDF and ADF content in multi-level ammonia treatment was thought to be due to high levels of urea used. Urea tends to reduce NDF content compared to other types of alkaline compounds [16,23,24]. The decreased NDF was suspected because of the ability of urea to bind with sugar compounds so that the NDF content that contains hemicellulose and cellulose will dissolve and result in a decrease in NDF content. Observation of dissolution of cotton cellulose compounds and wood fibers with urea/NaOH aqueous treatment using a microscope and viscometry showed swelling in the dissolution of these compounds [25] and starch compounds [26]. The ADF is part of the fiber fraction from the Van Soest analysis containing hemicellulose, lignin, cellulose, and silica. Several factors can cause a decrease in ADF content. The release of cellulose compounds due to the breakdown of cellulose compounds bound to lignocellulose and silica causes the cellulose compounds to dissolve with urea solution, which generally occurs. Polyorach and Wanapat [27], in their experiment, reported a decrease in the content of ADF treated with urea and its combination with Ca(OH)_2_ in rice straw. Nevertheless, several studies of feed treatment using urea tend to show the results of increased ADF content [28–31].

A large number of researches on digestibility have been carried out in measuring the quality of feed ingredients. A study to measure the digestibility of feed ingredients, such as forages, straw, and agricultural waste, has been carried out on its development. Straw, agricultural, and industrial waste products generally have a low digestibility value; thus, their use as animal feed is still deficient. Chemical treatments, such as urea, NaOH, and KOH compounds, have been carried out to increase the digestibility in the feed. Based on the results of many studies, such chemical treatments are proven to be able to improve digestibility [12,28–34].

Table 2 shows the DMD and OMD *in vitro*. High DMD has a positive correlation with OMD. This correlation is because OM is the main element of DM composition. Thus, increased DMD results in increased digestibility of OMD. The highest digestibility value was founded in multi-level ammonia treatment. The high digestibility was due to the high ammonia produced. Thus, accelerating the swelling of lignocellulosic and silica bonds, which are factors that cause low digestion of oil palm compressive fibers (PPF). Nitrogen derived from urea that seeps in the PPF can increase ammonia levels in the rumen. A substrate is available to improve the level and efficiency of protein synthesis by microbes [35]. Furthermore, ammonia derived from urea will increase the amount and activity of rumen microbes which results in increasing the DMD because of the more effective rumen work to degrade CF components during the fermentation process [9,16,23,24,36].

At the same time, because rumen microorganisms are difficult to digest, the high content of fiber components, such as NDF and ADF, in the control treatment is considered to be the cause of low DM digestibility. The NDF and ADF composition values in each treatment can be seen in Table 1. According to McDonald [37], cell wall components consisting of NDF, ADF, lignin, and silica are limiting factors in the degradation of food substances, mainly DM, OM, and CP in feed ingredients. PPF includes rough food (roughage), which is feed material that comes from plantation waste or crops that have been harvested. When viewed from the nutritional content, PPF has a low content and digestibility. Still, about 80% of potential substances can be digested and used for animals as an energy source [7,8].

The results of the total analysis of VFA and N-NH_3_ *in vitro* showed significant differences in the ammonia treatment. VFA concentrations shown at A-Mul had higher concentrations (*p* < 0.05) than control, but did not differ within 4% ammoniation treatment (*p* > 0.05). On the contrary, at N-NH_3_ concentrations, the highest concentrations were shown at 4% ammoniation treatment compared to the control treatment (*p* < 0.05). However, it was not significantly different from multi-level ammonia treatment (*p* > 0.05). The high concentration of VFA was caused by the dissolution of lignocellulosic bonds, which are covered by cell walls consisting of silica and lignin, which causes rumen microbes to penetrate the cell contents more quickly and produce VFA. This assumption was reinforced by the decrease in CF content and fraction of the constituent fibers that show descent with A-Mul treatment; each value can be seen in Table 1. VFA was generated from the fermentation of sugar compounds, which are usually found in plants in the form of cellulose and hemicellulose.

Cuissinat and Navard [25] stated that out of five modes of dissolution of wood fiber and cotton cellulose dissolved with an alkaline solution, cellulose will be in the third mode of experiencing a significant swelling with ballooning and partially dissolving the fiber, still maintaining the shape of the fiber. Based on this, it can be presumed that at least part of the fraction of the decomposed fiber causes during fermentation *in vitro*, rumen microbes with cellulase enzymes can optimally increase the production of VFA in the rumen.

Significant differences in N-NH_3_ concentrations were shown in the 4% ammonia treatment. However, this value is not different from multi-level ammonia treatment. This difference was thought to be due to urea used as a source of nitrogen during the PPF ammoniation process hydrolyzed to ammonia. Ammonia formed during the ammoniation process will be fixed into the PPF to increase the nitrogen content, ultimately increasing the rumen N-NH_3_ concentration. The nitrogen content of ammoniated PPF can be reflected in the increased protein content. An increase in protein occurs when there is an increase in the concentration of N-NH_3_ rumen fluid and the level of CP content is above 13%. Increasing CP content can be done by decreasing CF content. Wylie and Steen [38] state that treatment was required to reduce the fiber content of raw materials carried out by acid hydrolysis to produce high protein feed.

The more protein degraded by rumen microbes, the higher the production of N-NH_3_. Most rumen microbes require N-ammonia for growth. Zhao et al. [16] stated that ammoniation techniques could carry out the stretching of fiber fraction bonds and increase CP content, resulting in sufficient nitrogen for rumen microbial growth. Polyorach and Wanapat [27] stated that ammonia as the primary nitrogen source was essential for protein synthesis of rumen microorganisms. It is known that 80% of bacteria in the rumen used ammonia as the only source of nitrogen for growth [39,40].

Furthermore, Ørskov and Mcdonald [41] stated that N-NH_3_ production depended on the solubility of N from a feed ingredient, the amount of food protein, and food length in the rumen. The feed protein in the rumen is hydrolyzed into amino acids and oligopeptides by proteolytic enzymes. In addition, amino acids require further catabolism to form ammonia, VFA, and CO_2_. A specific range of ammonia concentration is required to maximize the rate of microbial protein synthesis.

## Conclusion

Based on the results described above, it can be concluded that multi-level ammoniation has a significant effect on the digestibility of feed ingredients with high fiber content and can shorten the curing period of the ammoniation process.

## List of Abbreviations

PPF, Palm press fiber; CP, Crude protein; EE, Ether extract; CF, Crude fiber; DM, Dry matter; OM, Organic Matter; NDF, Neutral Detergent Fiber; ADF, Acid Detergent Fiber; DMD, Dry matter Digestibility; OMD, Organic matter digestibility; VFA, Volatile Fatty Acids; AOAC, Association of Official Analytical Chemists.
